# pLannotate: engineered plasmid annotation

**DOI:** 10.1093/nar/gkab374

**Published:** 2021-05-21

**Authors:** Matthew J McGuffie, Jeffrey E Barrick

**Affiliations:** Department of Molecular Biosciences, Center for Systems and Synthetic Biology, The University of Texas at Austin, 2500 Speedway A5000, Austin, TX 78712, USA; Department of Molecular Biosciences, Center for Systems and Synthetic Biology, The University of Texas at Austin, 2500 Speedway A5000, Austin, TX 78712, USA

## Abstract

Engineered plasmids are widely used in the biological sciences. Since many plasmids contain DNA sequences that have been reused and remixed by researchers for decades, annotation of their functional elements is often incomplete. Missing information about the presence, location, or precise identity of a plasmid feature can lead to unintended consequences or failed experiments. Many engineered plasmids contain sequences—such as recombinant DNA from all domains of life, wholly synthetic DNA sequences, and engineered gene expression elements—that are not predicted by microbial genome annotation pipelines. Existing plasmid annotation tools have limited feature libraries and do not detect incomplete fragments of features that are present in many plasmids for historical reasons and may impact their newly designed functions. We created the open source pLannotate web server so users can quickly and comprehensively annotate plasmid features. pLannotate is powered by large databases of genetic parts and proteins. It employs a filtering algorithm to display only the most relevant feature matches and also reports feature fragments. Finally, pLannotate displays a graphical map of the annotated plasmid, explains the provenance of each feature prediction, and allows results to be downloaded in a variety of formats. The webserver for pLannotate is accessible at: http://plannotate.barricklab.org/

## Graphical Abstract

**Graphical Abstract:**
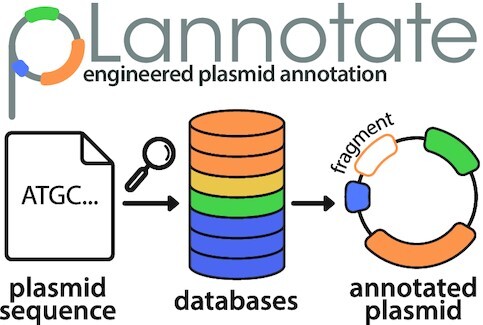
pLannotate is a web server for annotating engineered plasmids. It integrates predictions from multiple databases and detects fragments of features leftover from a plasmid’s history that may impact its function.

## INTRODUCTION

Engineered plasmids are widely used in the biological sciences for diverse applications that include amplifying DNA, overexpressing proteins, building genetic circuits and engineering metabolism. It has been nearly a half century since recombinant DNA technology was first developed and used to domesticate laboratory plasmids from their wild counterparts (1). For decades, engineered plasmids were primarily constructed by combining chunks of naturally occurring DNA sequences using restriction enzymes or integrases. Even now, when precision DNA synthesis and assembly methods are available, new plasmids typically incorporate pieces of existing plasmids.

Their circuitous cloning histories often mean that present-day maps describing what features are found on plasmids are imprecise and missing information. Plasmid backbones are especially prone to incomplete annotation, for example. A researcher may have a working knowledge of what origin of replication and selection markers a plasmid backbone contains without: (i) understanding precisely what promoters, proteins, and noncoding RNAs give it those functions; (ii) knowing where they are located in its sequence and (iii) realizing that it also encodes additional genetic elements with other functions. Remixed plasmids may also incorporate cryptic fragments of previously functional components that were carried along with other features on the DNA chunks used in plasmid construction. At best, such unannotated features and forgotten genetic elements take up valuable space on plasmids that have replication size limits. At worst, these fragments of functional sequences could have unintended consequences when they are inadvertently combined with new sequences. For all of these reasons, giving researchers access to higher-resolution plasmid maps has the potential to make genetic engineering projects more predictable, efficient, and reliable.

Computational tools that are currently available for annotating engineered plasmids have limitations. The most fully featured web server designed for this task, PlasMapper (2), can only annotate a set of 341 features and has not been updated for >15 years. Desktop programs for plasmid annotation, such as SnapGene, are commercial software and use proprietary algorithms. Furthermore, these and other existing tools have several shortcomings: they do not filter feature matches to resolve ambiguity and overprediction, their feature libraries are not well-documented, and they do not report incomplete features that may still impact plasmid function. On the other hand, pipelines intended for microbial genome annotation, such as Prokka (3), do not adequately identify key features that are specifically found in engineered plasmids. We created the pLannotate webserver to comprehensively annotate engineered plasmid features, including cryptic fragments.

## MATERIALS AND METHODS

### Databases

**Figure 1.**
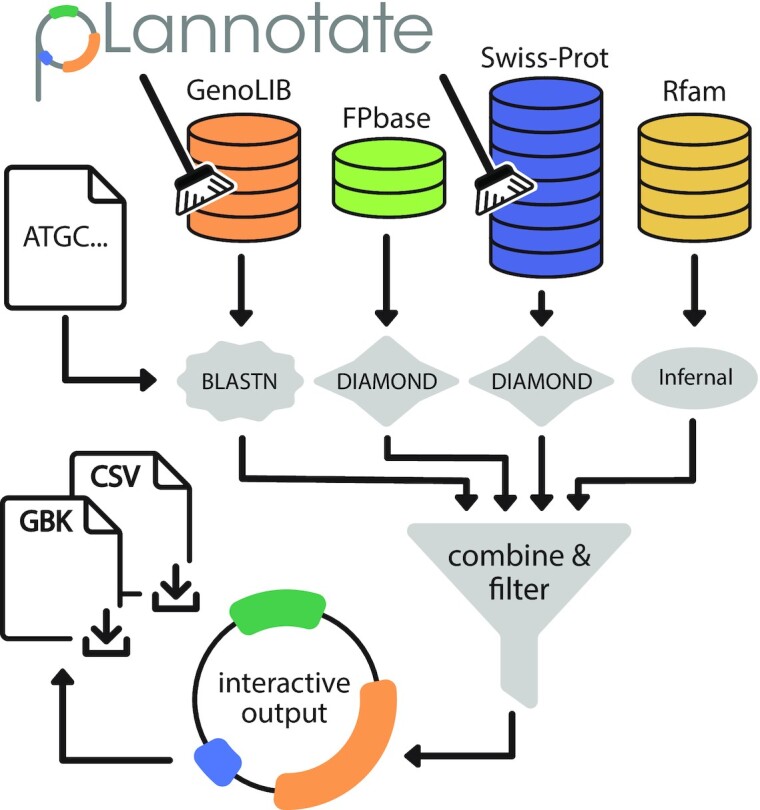
pLannotate web server workflow. Users input a plasmid sequence as a FASTA file, a GenBank file or raw text. This sequence is queried against various feature databases. Hits are compiled and filtered to ensure that the most informative matches are reported. Annotation results are displayed as a graphical plasmid map for users to interact with and can be download in different formats.

The overall workflow used by the pLannotate web server is illustrated in Figure 1. pLannotate currently utilizes four databases of genetic parts and proteins. First, we obtained the feature database used by SnapGene, which was first constructed as the GenoLIB biological part database (4). To check for completeness, we cross-referenced this list with 195 426 GenBank files downloaded from Addgene (5,6), a non-profit plasmid repository, that each contained automated annotations of plasmid features made by the SnapGene platform. These features were deduplicated by removing sequences that were identical to one other. This left 13 240 unique features with associated descriptions. These elements include promoters, terminators, selectable markers, origins of replication, and more. Many fluorescent protein variants with very different properties (e.g. spectra) have sequences that differ by only one or a few amino acids. In order to more precisely annotate fluorescent proteins, pLannotate uses FPbase (7), a comprehensive database of engineered fluorescent proteins that provides detailed histories and phenotypes for each sequence, as its second feature database. In order to detect a wide variety of other proteins, we also used the Swiss-Prot database (8), which currently contains more than half a million manually curated protein sequences. This third data source allows pLannotate to identify the many proteins from across all domains of life that may be inserted into engineered plasmids. To ensure accurate predictions, pLannotate only uses the subset of Swiss-Prot that has an Annotation Score of 3 or higher. This score threshold indicates that an entry has experimental evidence demonstrating the existence of a protein or that a sequence has convincing homology to a known protein. Fourth, pLannotate uses the Rfam database (9), which currently contains covariance models trained to detect 3940 families of noncoding RNAs.

### Annotation

In order to identify features in an input plasmid sequence, pLannotate uses different sequence homology search programs and parameters for each database (Table 1). Nucleotide BLAST (10) is used to query the GenoLIB features collected from Addgene. DIAMOND (11)—a faster implementation of BLAST specifically for proteins—is used for the FPbase and Swiss-Prot databases (Figure 1). While DIAMOND is slightly less sensitive than protein BLAST, the loss of sensitivity is not important since the aim is to detect only near perfect matches in order to accurately report the function of the protein. To further increase the chances of locating close protein homologs, the DIAMOND search is parameterized with the PAM30 matrix. Since DIAMOND is used to search translated nucleotide sequences against a protein database, this strategy enables pLannotate to also identify these proteins even when the sequences that encode them have been codon-optimized or contain other engineered silent mutations. GenoLIB protein sequences are identified using nucleotide BLAST instead of a protein BLAST or DIAMOND search, since different codon-optimized variants of proteins are found within this database and each may have a unique description. Covariance model searches using Infernal (12) are used to find matches to RNA families in Rfam.

**Table 1.** Databases and search programs used by pLannotate to identify plasmid features

Database	Number of features	Search program	*E*-value cutoff	Percent identity cutoff
**GenoLIB** (Compiled 10/29/20)	13,240	BLASTN	1	98%
**FPbase** (Downloaded 9/2/20)	762	DIAMOND	0.001	98%
**Swiss-Prot** (Release 2020_05, Annotation Score ≥3)	547,899	DIAMOND	0.001	10%
**Rfam** (Release 14.5)	3,940	Infernal	1	N/A

In order to properly annotate plasmids, most of which are circular, versus the linear nature of the input DNA strings, the plasmid DNA sequence is duplicated before performing searches. Hits found only within the second copy of the duplicated sequence region are discarded. Hits that span the boundary are reported after correcting their start and end coordinates. However, some types of plasmids are linear, such as the pJAZZ linear cloning vector (13), so pLannotate also includes an option for linear annotation. The algorithm for identifying hits in linear constructs is identical to the algorithm employed for circular constructs, except the input sequence is not doubled since there is no boundary to span.

After identifying potential features matching each database, many hits often overlap the same DNA bases of the plasmid. This is particularly true for features like promoters, which may have different canonical variants which can be longer or shorter than other related variants. This is a problem with engineered proteins as well, epitomized by fluorescent proteins which often only differ very slightly in sequence from one another. Typical BLAST statistics such as *E*-values and bit scores may not rank the most likely feature the highest due to differences in the lengths of matches and how homology is scored. Therefore, pLannotate uses a customized scoring system to determine which hits should be reported. All hits are given a score that is the product of three terms: (i) the length of the match, (ii) the percent sequence identity of the match and (iii) the fraction of the length of the feature in the database that the match covers. Since Rfam matches are reported when a covariance model score exceeds a prediction threshold and not due to a match to a specific feature, we assign them the maximum score of 100%. All hits are sorted by this score, and if a hit with a lower score is found within the bounds of a hit with a higher score, it is dropped. However, some valid features inherently have some overlap, such as the *repA* and *repC* genes in the RSF1010 plasmid origin of replication (14). To allow for some overlap, lower-scoring matches are only removed if they intersect the region covered by a higher-scoring match after trimming away 15% of its total length on each side. After filtering, hits that cover less than 95% of the length of the feature in the database are marked as possible feature fragments.

### Implementation

pLannotate is written in Python3 and uses the Streamlit open-source library for creating web apps. Files are parsed and written using Biopython. The interactive plasmid map is generated using the Bokeh plotting library. The pLannotate webserver is hosted on Amazon Web Services.

## WEB SERVER USAGE

### Input

pLannotate accepts a FASTA or GenBank file upload or a DNA sequence entered in a text box. Sequences should be represented using IUPAC nucleotide codes: A,T,G,C, plus nucleotide ambiguity codes (15). pLannotate will prevent users from submitting sequences containing invalid bases as well as multi-sequence files, malformed files, files larger than 1 MB, and sequence inputs larger than 50,000 bases. Example plasmid sequences are also provided. Plasmid annotation takes a matter of seconds, so there is no need for user registration or a job queuing system.

### Output

Output is displayed as an interactive plasmid map, allowing the user to zoom in and out with their mouse scroll wheel, pan by clicking and dragging, and view information about an annotation by hovering their mouse over a feature of interest. Features are denoted by arrows, the tips of which indicate the direction of the feature. The annotations that appear on hover in the plasmid plot contain the name, type, and description of the feature, as well as a combined score equal to the fraction of the length of the feature matched multiplied by the percent identity of the match. Features that are identified by pLannotate to be reasonably complete (with some tolerance for mismatches/indels) are denoted by arrows with black outlines that are filled with colors indicating different feature types and databases. Annotations of predicted feature fragments (<95% match length) are denoted by white arrows outlined in the same colors.

Features are also displayed in a table below the interactive plasmid map. Descriptive information about the function of the feature as well as information about the coverage and the percent identity of the match is provided. Output from pLannotate can be downloaded as a GenBank flat file or a comma separated values (CSV) text file. When annotating an uploaded GenBank file, users also have the option to download a GenBank file that merges pLannotate predictions with annotations present in the original file so that they can be compared.

## RESULTS AND DISCUSSION

### Case studies

Given its expanded databases and ability to identify partial feature matches, pLannotate should produce more complete plasmid annotations than other tools. To see whether this was the case, we examined pLannotate predictions in detail for three different plasmids and compared them to annotations produced by the PlasMapper web server and the SnapGene Viewer desktop program.

#### Case study 1

**Figure 2.**
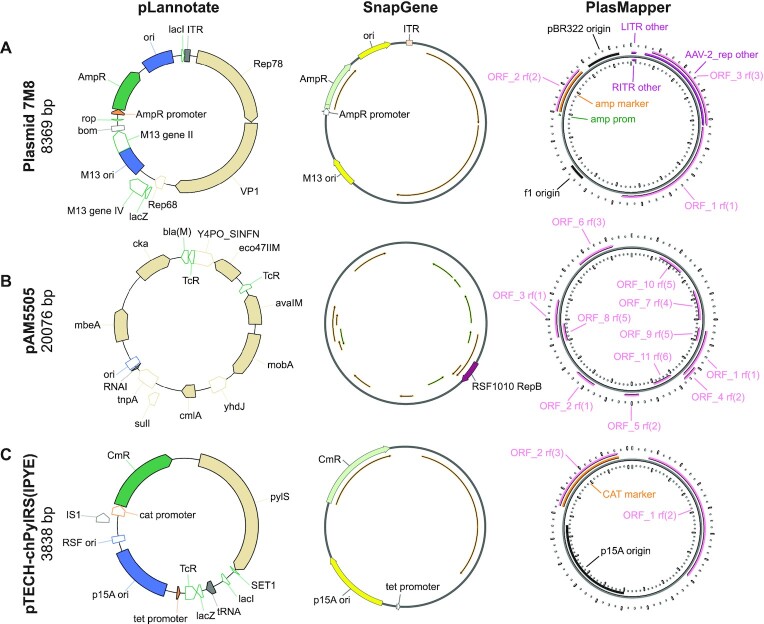
Annotation results for three engineered plasmids. Each panel compares output from the pLannotate web server, the SnapGene viewer desktop program, and the PlasMapper web server. SnapGene and PlasMapper both have options to display generic open reading frames above a length cutoff that do not match specific features in their databases. These ORFs are displayed as thin arrows for SnapGene results and pink arrows for PlasMapper results. pLannotate displays features in different colors based on their type if they are matches to the GenoLIB feature database and in tan if they are matches to proteins in Swiss-Prot. Fragmentary matches are shown as unfilled arrows. Labels were manually edited to improve legibility for all tools. (**A**) Plasmid 7M8 is used for adeno-associated virus (AAV) vector production. (**B**) Plasmid pAM5505 is a helper plasmid for engineering cyanobacteria via conjugal transfer of DNA. (**C**) Plasmid pTECH-chPyIRS(IPYE) contains an engineered aminoacyl-tRNA synthetase enzyme. Note the partial TcR open-reading frame downstream of the tet promoter that is discussed in the text

The 7M8 plasmid for adeno-associated virus (AAV) vector production (Addgene #64839) was constructed by Dalkara *et al.* (16). It contains a mutant adenovirus capsid protein generated by directed evolution. The two full-length adenovirus proteins in this plasmid are detected as generic open reading frames by both SnapGene and PlasMapper (Figure 2A), and PlasMapper further labels one as an AAV-2 replication protein. pLannotate more fully identifies both adenovirus genes based on matches to Swiss-Prot, including the experimentally evolved capsid gene, which it identifies correctly as the VP1/AAV2 capsid protein. pLannotate identifies this protein as an imperfect 97% match against the reference, which suggests that it has been modified. pLannotate also correctly identifies the AAV initiator protein, Rep78, contained on the plasmid and finds a 183 bp fragment of the AAV *rep68* gene, that is likely left over from the cloning process. Additionally, pLannotate finds multiple incomplete feature matches in the backbone of the plasmid that are common to many engineered plasmids, including fragments of *lacZ*, *lacI* and phage M13 genes II and IV. These feature fragments were likely incorporated into the plasmid backbone long before this specific plasmid was constructed. This case study demonstrates pLannotate's ability to identify partial features as well as proteins and protein variants not found in databases of standard plasmid features.

#### Case study 2

The pAM5505 plasmid (Addgene #132664) constructed by Elhai *et al.* is a helper plasmid for conjugal transfer of DNA (17). It was developed for genetically engineering cyanobacteria. This plasmid is poorly annotated by SnapGene and needs to be trimmed by 76 bases to be annotated by PlasMapper since it exceeds its sequence size limit of 20 000 bases (Figure 2B). SnapGene only predicts a single gene, *repB*, though this is an incorrect annotation, as the full gene—which is correctly found by pLannotate—is actually over twice as long as the SnapGene annotation. pAM5505 contains sequences that are rare in plasmids that are only used in *Escherichia coli*, including proteins for mobilizing plasmids with an origin of transfer and three methyltransferases that protect plasmid DNA from restriction enzymes found in cyanobacteria (18). All of these genes are identified by pLannotate. pLannotate further finds a complete RNAI sequence (the anti-sense regulator of the ColE1 origin), which is adjacent to a partial match of the ColE1 origin from *E. coli* plasmid pBR322. These elements are likely from the pBR322 origin that was combined with a pDU1 cyanobacterial origin of replication to create the shuttle vector backbone (19) that was used in constructing pAM5505. Lastly, pLannotate finds partial β-lactamase (*bla*) and the tetracycline resistance (*tcR*) genes that are likely also derived from pBR322. This case study highlights pLannotate's improved ability to decipher the sequences present on plasmids that have complex cloning histories and include less-common genetic parts. Database coverage, especially of parts used outside of *E. coli*, is still an area for future improvement, as pLannotate does not annotate the pDU1 cyanobacterial specific origin of replication in pAM5505.

#### Case study 3

The pTECH-chPylRS(IPYE) plasmid (Addgene #99222) constructed by Bryson *et al.* (20) is part of a set of plasmids that each contains an aminoacyl-tRNA synthetase variant created by directed evolution. These genes are annotated by pLannotate but are simply labelled as generic ORFs by SnapGene and PlasMapper (Figure 2C). pLannotate also identifies the tRNA contained within the plasmid, which neither SnapGene nor PlasMapper locates. The common backbone for these plasmids, pTECH, contains a *tet* promoter that is found by pLannotate and SnapGene, but not PlasMapper. There is also a truncated tetracycline resistance gene, which is only detected by pLannotate, located immediately downstream of this promoter. This *tet* promoter is likely to still be active. Translation of the resulting mRNA would begin at the start codon of the truncated ORF and continue until encountering a stop codon. In this instance, it would produce a 102-residue protein that fuses together amino acids encoded by the first part of the tetracycline resistance gene coding sequence and the noncoding strand of a fragment of *lacZ*. Inadvertent translation of proteins from noncoding regions, like this one, can potentially result in toxicity to the host cell due to a number of mechanisms. For example, these atypical proteins may alter host gene expression (21) or form inclusion bodies (22), which can lead to decreased fitness and cell death (23). This case study highlights how more complete plasmid annotation by pLannotate can discover potential problems caused by cryptic genetic elements.

Overall, these case studies demonstrate pLannotate's unique strengths and show that it annotates a much larger fraction of each plasmid's DNA sequence than SnapGene or PlasMapper. Additional examples of plasmid annotation results are available on the pLannotate website.

### Prevalence of feature fragments

**Figure 3.**
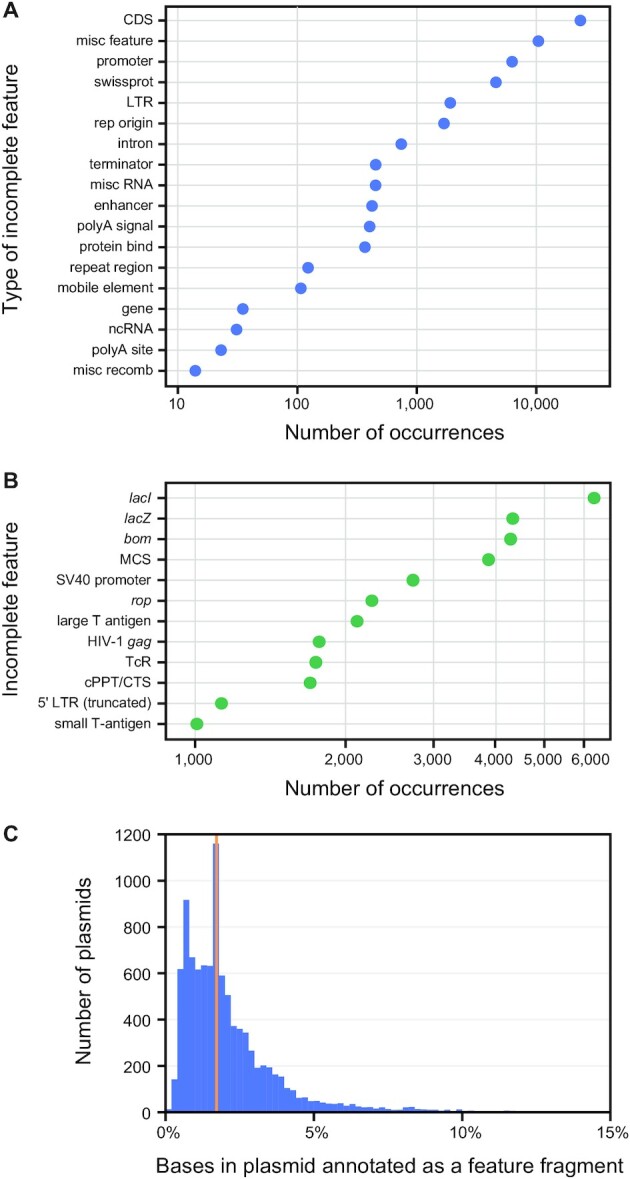
Incomplete fragments of genes and other genetic parts are common in engineered plasmids. We used pLannotate to annotate the sequences of 10,000 plasmids that are available from Addgene and analyzed predictions of putative feature fragments. (**A**) Overall abundance of fragments derived from different types of features. Protein coding regions are divided into the CDS category for matches to the GenoLIB or FPbase databases and the swissprot category for matches to the Swiss-Prot database. (**B**) Identities of the most common feature fragments. (**C**) Histogram of the percentage of the bases in each plasmid that are derived from predicted feature fragments. The orange line at 1.7% represents the median.

To better understand how widespread leftover fragments of genes and genetic parts are on engineered plasmids, we used pLannotate to analyze the sequences of 10,000 plasmids that are available from the Addgene repository. Over 98% of these plasmids contain at least one prediction of a feature fragment. Most of these are incomplete pieces of protein coding sequences (CDSs) from the GenoLIB database (Figure 3A). These CDS fragments could be translated leading to unintended consequences or potential toxicity to cells if they are downstream of promoters as highlighted in *Case study 3*. Many of the most common features that are found as fragments are from early cloning vectors (Figure 3B). The *bom*, *rop*, and *tcR* genes are all elements found on one of the first engineered plasmids, pBR322 (1). Others, including *lacI*, *lacZ* and the multiple cloning site (MCS), are found on pUC19 (24), a commonly used plasmid derived from pBR322. Of the plasmids found to contain at least one fragment, most had about 5% or less of the plasmid sequence occupied with fragmented features with the median value of 1.7% (Figure 3C). Thus, the presence of these incomplete genetic relics typically does little to increase the size of a typical plasmid. However, their continued presence on many plasmids is a potential landmine for unsuspecting researchers who may combine these plasmids in new ways and inadvertently activate their latent functions.

## CONCLUSION

The pLannotate web server updates and improves upon existing tools that are freely accessible for annotating engineered plasmids. pLannotate uses multiple feature databases to more comprehensively annotate plasmids or other engineered DNA constructs, and it finds fragments of functional components so that their potential effects on plasmid function can be considered. pLannotate annotates plasmids in seconds, displays results in a traceable and interactive output format, and provides users with several ways to download and interact with the predictions. The GenBank output from pLannotate can be input into a variety of DNA design programs for viewing and editing the annotation results, but this format has some limitations. Synthetic Biology Open Language (SBOL) (25) offers a richer vocabulary for describing features and their interactions. In the future, support for SBOL output could allow pLannotate to better integrate with synthetic biology design workflows, including tools that use standardized glyphs to visualize the features and functions of plasmids. Another limitation is that existing databases of engineered DNA parts—including the Registry of Standard Biological Parts (26), the JBEI-ICE registry (27) and the GenoLIB set used by pLannotate—lack the completeness and expert curation of databases such as FPbase and Rfam. There is an opportunity to improve these databases in conjunction with future development of pLannotate so that it can provide richer and more precise annotations, e.g. of specific types of *E. coli* plasmid origins of replication in which known point mutations and accessory factors control copy number. Since the code driving pLannotate is open source, we have created a platform that can be replicated, updated, and improved upon by the scientific community to better inform plasmid engineering projects.

## DATA AVAILABILITY

The source code for pLannotate is available on GitHub (https://github.com/barricklab/pLannotate) under the GNU General Public License version 3. Feature databases used by the public webserver are provided in the GitHub repository or are freely accessible from the Uniprot Consortium or Rfam.
